# *Kerstersia gyiorum* infection in a patient with chronic suppurative otitis media identified by whole genome sequencing: a case report

**DOI:** 10.1590/S1678-9946202567049

**Published:** 2025-08-18

**Authors:** Daofu Shen, Hongmei Niu, Wu Zhao, Mingquan Shang, Hao Yu, Xiaohong Wang, Fuqian Zhao, Lei Wang

**Affiliations:** 1 Chifeng Municipal Hospital, Department of Clinical Laboratory, Chifeng, Inner Mongolia, People’s Republic of China; 2 Hubei University of Chinese Medicine, Hubei Shizhen Laboratory, Wuhan, Hubei Province, People’s Republic of China

**Keywords:** Chronic suppurative otitis media, Kerstersia gyiorum, MALDI-TOF MS, Whole genome sequencing, Antimicrobial therapy

## Abstract

A 52-year-old female patient suffering from chronic suppurative otitis media had a bacterial strain isolated from her ear swab specimens, which could not be identified using conventional methods, but it was eventually identified as *Kerstersia gyiorum* via whole genome sequencing (WGS). Antimicrobial drug susceptibility testing was conducted on the isolated strain and the results demonstrated its susceptibility to a range of antimicrobial drugs, including ofloxacin, ceftriaxone, and other agents. However, resistance to chloramphenicol was observed. The patient was treated with ofloxacin, ceftriaxone, and dexamethasone, resulting in an improvement in symptoms. This report describes the first documented case of *Kerstersia gyiorum* causing chronic suppurative otitis media in China. WGS provided definitive species identification in which conventional methods failed, demonstrating its critical role in diagnosing atypical pathogens.

## INTRODUCTION

*Kerstersia gyiorum* is a Gram-negative bacterium found in various animals, including humans, in which it has been associated with various infections^1^. *Kerstersia gyiorum* belongs to the genus *Kerstersia* in the family *Alcaligenaceae*, which includes *K. gyiorum* and *K. similis* ^2^. This genus, which is originally described by Coenye *et al*.^3^ and classified within the family *Alcaligenaceae* alongside *Alcaligenes*, *Achromobacter*, *Bordetella*, and *Pigmentiphaga*, bears the specific epithet ‘gyiorum’ (Latin for ‘from the limbs’), denoting its isolation source from limb wound swabs. The genus *Kerstersia* is oxidase negative and has no specific odor. As a new member of the alkali-producing family, *K. gyiorum* has been isolated from wounds (lower leg and right foot)^4,5^, respiratory tract^6^, urine specimens^7^, bacteremia^8^, stasis dermatitis^9^, soft tissue biopsy samples^10^, and is most commonly found in chronic suppurative otitis media (CSOM)^11^ and its incidence has increased recently. In China, *Kerstersia gyiorum* has been reported to be isolated from sputum samples of infected patients^4^, but there are no reports on the isolation of *Kerstersia gyiorum* from CSOM samples.

### Ethics

This study was reviewed and approved by the Ethics Committee of Chifeng Municipal Hospital. This subject strictly abides by the Declaration of Helsinki, and all individuals participating have given informed consent.

## CASE REPORT

**Figure 1:**
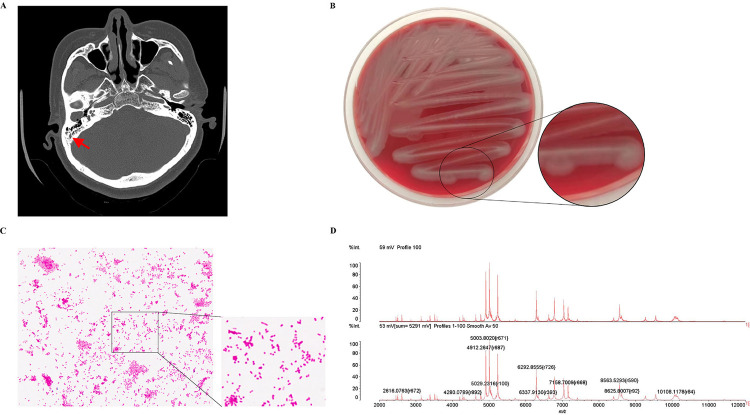
Non-contrast computerized tomography (CT) scans reveal the presence of otomastoiditis. Morphological characteristics and mass spectrometry image of Kerstersia gyiorum: (A) Planar bone window CT images to visualize the condition; (B) The colony morphology of Kerstersia gyiorum; (C) The result of Gram staining microscopy of Kerstersia gyiorum (1000×); (D) Mass spectrometry image of Kerstersia gyiorum.

A 52-year-old female patient went to the outpatient clinic of Chifeng Municipal Hospital complaining about dizziness, accompanied by a history of fluid accumulation in the external ear canal for several years and impaired hearing. The patient denied smoking but acknowledged a history of alcohol consumption and she did not have a history of systemic diseases. According to her complaints, she had treated herself with various ear drops and intermittent erythromycin ointment. Physical examination revealed bilateral tympanic membrane invagination, poor motility, and no fluid level, and CT examination revealed opacification of the right middle ear and mastoid (Figure 1A). The primary care physician made the following clinical diagnosis: chronic purulent otitis media. To identify the pathogen, the primary care physician requested a general bacterial culture and identification (ear swab) for the patient. The patient’s ear swab specimens were then inoculated by zonal delineation onto Columbia blood, MacConkey, and chocolate plate Petri dishes (Zhengzhou Humanwell Biocell Biotechnology Co., Ltd) and incubated for 18-24 h at 37 °C in a CO_2_ incubator. Pure colonies of two different species (one grows sparingly, the other dominantly) were then isolated, cultured, and identified by matrix assisted laser desorption ionization-time of flight mass spectrometry (MALDI-TOF MS, bioMérieux, Marcy l’Etoile, France). The fractionated pure colonies (bacteria that grow sparingly) were identified as *Alcaligenes faecalis* subsp. faecalis (99% confidence), whereas the other (bacteria that grow dominantly) could not be identified (see Figure 1D for mass spectral pattern). The predominant strains demonstrated robust growth on blood, MacConkey, and chocolate agar plates. The strains cultivated on blood agar plates showed an irregular surface, lacked hemolysis, and displayed grayish-white, moist colonies with irregular, scalloped, serrated margins (Figure 1B). A pure culture of the isolated strain was collected for Gram staining and observed under a standard light microscope. Gram staining revealed Gram-negative bacteria, manifesting as either pink, non-uniform bulbous, or rod-shaped (Figure 1C). Subsequent attempts to identify the species using the Vitek 2-Compact GN identification card (bioMérieux, Marcy l’Etoile, France) met with failure, as the Vitek 2-Compact analysis system failed to recognize it. Further research is necessary to identify these bacteria and obtain more information about it. Prior to commencing the bacterial culture, the physician administered ear drops containing ofloxacin and dexamethasone, in addition to 2 g/day of ceftriaxone administered intramuscularly for a total duration of ten days. The patient’s ear discharge ceased after the conclusion of the treatment, and she was instructed to continue using ofloxacin- dexamethasone drops. At a subsequent follow-up, she reported that her ear symptoms had improved significantly.

Pure bacteria were isolated and sent to Shanghai Applied Protein Technology Co., Ltd. for genomic DNA extraction and whole genome sequencing (WGS), Thus, the bacterium was identified as *Kerstersia gyiorum*. The minimal inhibitory concentration (MIC) values of the bacteria against 20 antibiotics were then determined by the E-test method according to the criteria for bacteria of the non-enterobacterial order of the Clinical and Laboratory Standards Institute^12^. Table 1 shows the results of the antimicrobial susceptibility testing of *Kerstersia gyiorum*.

**Table 1:** - The results of antibiotic susceptibility tests.

Antimicrobial agent	MIC (μg/mL)	Interpretation
Cefepime	4	S
Ceftazidime	1	S
Ceftriaxone	0.25	S
Cefotaxime	1	S
Ofloxacin	0.5	S
Ciprofloxacin	2	S
Levofloxacin	1	S
Trimethoprim-Sulfamethoxazole	0.125	S
Piperacillin	0.5	S
Piperacillin-Tazobactam	0.5	S
Ticarcillin-Clavulanate	0.064	S
Aztreonam	8	S
Imipenem	0.5	S
Meropenem	0.016	S
Gentamicin	1	S
Minocycline	0.5	S
Doxycycline	1	S

S = Susceptible.

**Figure 2:**
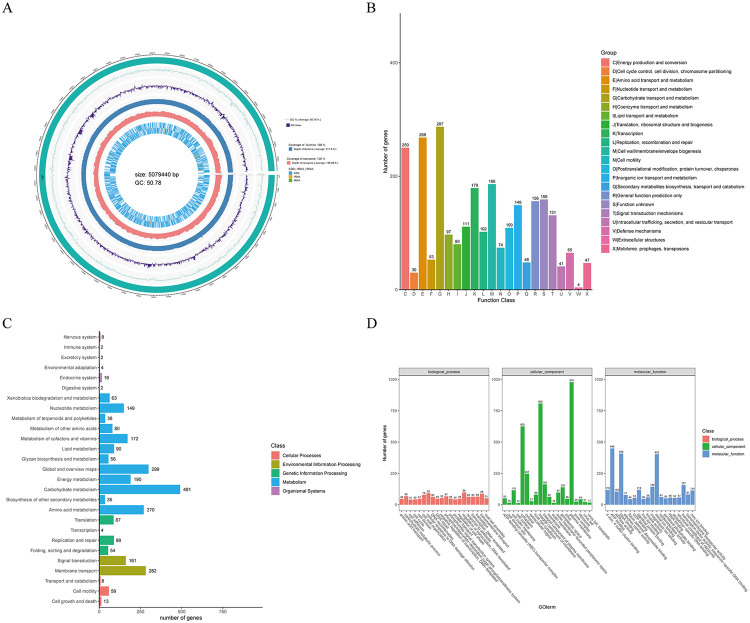
Results of whole genome sequencing analysis of *Kerstersia gyiorum*: (A) Genomic map of Kerstersia gyiorum. The outermost circle of the picture is the position coordinates of the genome sequence, showing the distribution of annotation results for coding gene and functional genes from outside to inside. (B) COG function classification diagram of the Kerstersia gyiorum genome; (C) Classification diagram of KEGG metabolic pathway of the Kerstersia gyiorum genome; (D) GO function classification map of the Kerstersia gyiorum genome.

The complete genome of *Kerstersia gyiorum* is 507,9440 bp in length, with a G+C% content of 50.78%, and contains a total of 5,219 predicted genes, 99 tRNA and 22 rRNA genes, which are illustrated in Figure 2A. Figure 2B illustrates the COG functional annotations, whereas the GO and KEGG pathway enrichment analysis results are shown in Figures 2C and 2D, respectively.

Annotation of resistance genes using the Comprehensive Antibiotic Resistance Database^13^ revealed the presence of several resistance genes in the *Kerstersia gyiorum* genome. Of these, 16 Perfect Hits were identified (mdtE, AcrE, emrB, emrR, evgA, emrY, marA, H-NS, mdtH, mdtG, msbA, *Escherichia coli* acrA, acrB, ampH, FosA7.5, and cpxA), including targeted resistance to several classes of drugs, including fluoroquinolones, cephalosporins, tetracyclines, macrolides, coumarin antibiotics, and rifamycins, as well as genes associated with multiple drug-resistant efflux pumps.

## DISCUSSION

*Kerstersia gyiorum* is a rare microorganism that has been isolated from CSOM in previous case reports. It is challenging to distinguish *Kerstersia gyiorum* from other microorganisms using traditional biochemical methods, which is related to the fact that it shows similar characteristics to other microorganisms. Automated bacterial identification methods, such as the Vitek2 system, were also unable to accurately identify *Kerstersia gyiorum*. Furthermore, MALDI-TOF MS, which that has been widely implemented in microbial identification, was unsuccessful in this case. The inability to identify *Kerstersia gyiorum* may be attributable to the utilization of instrumentation from disparate manufacturers, which may have resulted in it not being included in the database of the instrument. However, recent reports have indicated that 16S rRNA gene sequencing has contributed to the identification of such rare bacteria. WGS is also a valuable approach if the objective is to obtain more information about the bacterial genome.

In contrast to most of the previous case reports, *Kerstersia gyiorum* in this case showed substantial discrepancies regarding antibiotic resistance. Previous cases of *Kerstersia gyiorum* typically demonstrated resistance to fluoroquinolone antibiotics, such as ciprofloxacin and levofloxacin^6,9,10^. Other studies have reported resistance to amitranam, fosfomycin, and trimethoprim-sulfamethoxazole^9^. In contrast, this case demonstrated susceptibility to all antibiotics. Previous case reports utilized cephalosporins or fluoroquinolones for the treatment of *Kerstersia gyiorum* infections, and antibiotics such as piperacillin-tazobactam, imipenem, or meropenem for the treatment of patients with severe mixed infections^14^. Consequently, as indicated by the drug susceptibility results, this patient had a wide choice of therapeutic agents at their disposal.

The development of molecular and genetic identification methods has led to the detection of new and rare bacteria in clinical specimens, thus contributing to the advancement of clinical microbiology. Whole genome sequencing technology is gradually emerging as an additional potent tool for microbial identification. We conducted a WGS and bioinformatics analysis of *Kerstersia gyiorum*. Prediction of resistance genes was facilitated, and we found that the bacterium have targeted resistance to fluoroquinolones, cephalosporins, tetracyclines, macrolides, coumarin antibiotics, and rifamycins. In addition, genes associated with multiple drug-resistant efflux pumps were identified. The results of GO and KEGG analyses were also relatively consistent. Compared to other WGS analysis of *Kerstersia gyiorum* ^6^, while core virulence and resistance mechanisms are conserved, genomic divergence (size, GC%, accessory genes) highlights strain-specific adaptations. These differences may reflect niche-specific evolution in clinical environments. Further phylogenomic analysis is necessary to clarify taxonomic and functional distinctions. As a relatively rare bacterium, *Kerstersia gyiorum* has been periodically reported in recent years; however, its pathogenic mechanism, inflammatory response, and effective prophylactic and therapeutic methods still need further investigation.

## CONCLUSION

Although chronic suppurative otitis media caused by *Kerstersia gyiorum* is a rare infection, it is a serious condition that, if left untreated, may result in the formation of cholesteatomas, which can lead to tympanic membrane perforation and other serious consequences. The patient’s prolonged failure to receive appropriate treatment delayed the condition’s progression; however, the administration of antimicrobial therapy led to a remission of the disease. The combination of ofloxacin and ceftriaxone was an effective treatment for tympanitis. The process of making a pathogenetic diagnosis was challenging, due to the rarity of the bacterium responsible for the patient1s chronic suppurative otitis media, which could not be identified by traditional methods. Whole genome sequencing was employed to successfully identify the bacterium. Whilst MALDI-TOF MS and 16S rRNA are conventionally employed when dealing with rare bacteria, whole genome sequencing is an effective method when a more detailed understanding is required.
